# Effect of Graft Choice for ACL Reconstruction on Clinical Outcomes in Combined ACL and MCL Injuries: Comparison Between Bone-Patellar Tendon-Bone and Hamstring Autografts

**DOI:** 10.3390/jcm13216316

**Published:** 2024-10-22

**Authors:** Kwangho Chung, Hyeongwon Ham, Sung-Hwan Kim, Young-Jin Seo

**Affiliations:** 1 Department of Orthopaedic Surgery, Yongin Severance Hospital, Yonsei University College of Medicine, Yongin 16995, Republic of Korea; khchung85@gmail.com; 2 Department of Orthopaedic Surgery, Severance Hospital, Yonsei University College of Medicine, Seoul 03722, Republic of Korea; iys03016@yuhs.ac; 3 Department of Orthopaedic Surgery, Gangnam Severance Hospital, Yonsei University College of Medicine, Seoul 06273, Republic of Korea; orthohwan@gmail.com; 4 Department of Orthopaedic Surgery, Dongtan Sacred Heart Hospital, Hallym University College of Medicine, Hwaseong 18450, Republic of Korea

**Keywords:** anterior cruciate ligament, graft choice, medial collateral ligament, bone-patellar tendon-bone, hamstring

## Abstract

**Background/Objectives:** The optimal graft, particularly in combined anterior cruciate ligament (ACL) and medial collateral ligament (MCL) injuries, remains controversial. We evaluated the influence of graft choice between bone-patellar tendon-bone (BPTB) and hamstring autografts on clinical outcomes in combined ACL and MCL injuries. **Methods:** This retrospective analysis included patients with concurrent ACL and MCL injuries who underwent single-bundle ACL reconstruction with BPTB (group B) or hamstring (group H) grafts, between 2010 and 2019, with a ≥2-year follow-up. Patients were classified based on the MCL injury grade (I, II, or III). Clinical outcomes were assessed through knee stability evaluations using valgus stress radiographs and the KT-2000 arthrometer, patient-reported outcomes using the International Knee Documentation Committee (IKDC) subjective score and Lysholm score, and radiologic outcomes using the IKDC radiographic grade. **Results:** The study included 169 patients (group B, 92; group H, 77). No significant between-group differences in knee stability or functional outcomes were found after follow-up. Within the same MCL injury grade, particularly in high-grade MCL injuries, BPTB grafts resulted in significantly better medial stability (side-to-side difference in medial joint opening on valgus stress radiographs: grade II, *p* = 0.006; grade III, *p* = 0.039) and functional outcomes (IKDC subjective score: grade II, *p* = 0.045; grade III, *p* = 0.038) than hamstring grafts. In the hamstring group, higher-grade MCL injuries were associated with worse outcomes (Lysholm knee score, *p* = 0.009; IKDC subjective score, *p* = 0.015). **Conclusions:** Graft choice in ACL reconstruction with concomitant MCL injuries may affect clinical outcomes, particularly in high-grade MCL injuries. Although both graft types performed similarly overall, BPTB grafts provided superior medial stability and functional results in higher-grade MCL injuries. However, caution is needed when interpreting these results due to limitations such as the small sample size and the lack of randomization in graft selection.

## 1. Introduction

Anterior cruciate ligament (ACL) rupture is often accompanied by other ligamentous injuries. The medial collateral ligament (MCL) is the most frequently damaged ligament among concomitant ligament injuries. The incidence of MCL injury has been reported to range from 16% to 38% of ACL rupture cases [1,2,3,4]. Conservative therapy is the mainstay of treatment for most MCL injuries, other than some cases such as displaced bony avulsion of MCL and trapped MCL rupture [5,6]. However, the typical treatment option for ACL ruptures with symptomatic instability is surgical reconstruction [7]. The results of ACL reconstruction in conjunction with non-operative MCL treatment in combined ACL and MCL injuries were reported to be successful in terms of knee stability, subjective functional outcome, and return to preinjury activity [5,8,9,10,11,12].

Graft selection is an influential factor in the outcomes of ACL reconstruction. Biomechanical properties, including the ultimate load to failure and graft stiffness, differ between graft sources [13,14,15,16]. Furthermore, complications related to donor-site morbidity are distinct for each graft. A bone-patellar tendon-bone (BPTB) graft can cause anterior knee pain and weakness of the quadriceps muscle [17,18]. Likewise, a hamstring graft has been reported to be related to weakness in hip extension and knee flexion [19,20]. The stability, functional outcomes, and graft failure rate after ACL reconstruction can be affected by the type of graft [20,21,22,23]. Nonetheless, the optimal choice of graft for ACL reconstruction remains a subject of debate. Moreover, discussions on the adequate graft for ACL reconstruction in combined ACL and MCL injuries in the literature have been sparse. According to the results of recent biomechanical studies [24,25], harvesting hamstring tendons for ACL reconstruction could lead to increased valgus instability in those with a concomitant MCL injury. Thus, the absence of a hamstring, in addition to MCL insufficiency, could deteriorate medial stability and affect surgical outcomes. However, to the best of our knowledge, no clinical study has been conducted on the effect of hamstring harvest on the result of ACL reconstruction in terms of knee stability and functional outcomes in patients with acute combined MCL and ACL injuries, especially considering the extent of MCL injury.

This study aimed to evaluate the influence of graft choice between BPTB and hamstring autografts on the clinical outcomes in combined ACL and MCL injuries and to find an appropriate graft for ACL reconstruction in such injuries. We hypothesized that hamstring harvest for ACL reconstruction in combined ACL and MCL injuries would adversely affect instability and functional outcomes and that BPTB autografts would provide superior results in these combined injuries compared to hamstring grafts.

## 2. Materials and Methods

### 2.1. Patients

After obtaining approval from the institutional review board of our institution, we reviewed the data of patients who had undergone ACL reconstruction between January 2010 and December 2019. We included patients according to the following criteria: (1) concurrent ACL and MCL injury without other ligamentous injuries, (2) single-bundle ACL reconstruction using autogenous BPTB or hamstring graft, and (3) a minimum follow-up of 24 months. We excluded the patients with (1) surgical treatment for MCL injury, (2) chondral lesion greater than Outerbridge grade 2 at the time of surgery, (3) disabled hoop function due to subtotal or total meniscectomy, (4) revision ACL reconstruction, (5) contralateral ACL reconstruction, (6) an operative history of the affected knee, and (7) malalignment of the lower extremity (normal mechanical axis line passes 8 ± 7 mm medial to the center of knee joint line on standing hip-knee-ankle radiographs) [26].

**Figure 1:**
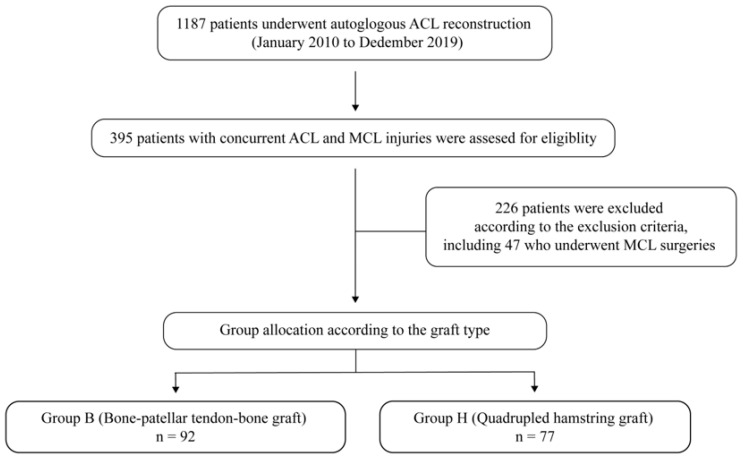
Flowchart of patient selection for the study.

**Figure 2:**
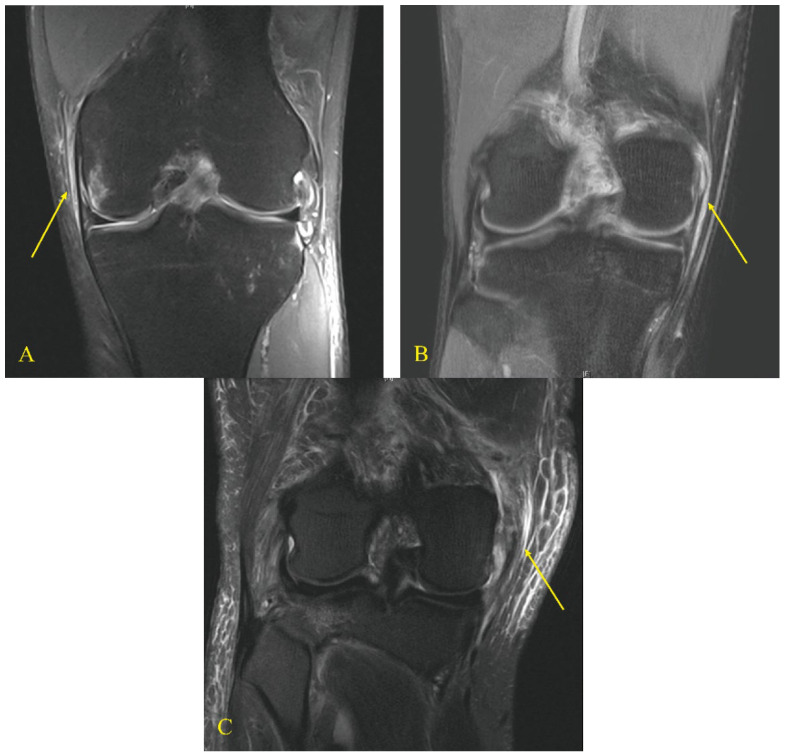
Grades of MCL injury on magnetic resonance imaging scans. Grade I (**A**) peri-ligamentous edema around the MCL on fluid-sensitive sequences, grade II (**B**) partial disruption of the ligamentous structures, and grade III (**C**) complete disruption of the superficial and deep MCL. Yellow arrows indicate the torn MCL based on the grade.

A total of 1187 patients underwent autologous ACL reconstruction during the study period. Of these, 169 patients were included in the study (Figure 1). As per the graft type used in ACL reconstruction, the patients were divided into two groups: group B, ACL reconstruction using a BPTB graft (n = 92) and group H, ACL reconstruction using quadrupled hamstring graft (n = 77). Each group was subclassified according to the grade of MCL injury on magnetic resonance imaging (MRI) scans [27]: grade I included peri-ligamentous edema around the MCL on fluid-sensitive sequences; grade II included partial disruption of the ligamentous structures; and grade III included complete disruption of the superficial and deep MCL (Figure 2) [27].

Graft selection was not randomized. However, we followed our own criteria for selecting graft materials [28]. If the length of the patellar tendon on MRI scan was <4 cm, a BPTB graft was selected. A hamstring graft was selected if the patellar tendon length was >4 cm to avoid a graft length mismatch.

The surgical procedure began with graft harvest. For BPTB grafts, the patellar bone block adjoining the patellar tendon was harvested. For hamstring grafts, the semitendinosus and gracilis tendons were harvested. After harvesting the graft, tibial and femoral tunnels were created on the footprints of the ACL in the respective graft diameters. The graft was then placed and secured at the tibial and femoral tunnels. All patients underwent the same rehabilitation protocol. Tolerable weight-bearing and knee motion were allowed immediately after surgery. Jogging, cycling, and swimming were permitted after 12 weeks. Sports activity involving pivoting, jumping, or sidestepping was allowed after 6 months.

### 2.2. Clinical Assessments

Clinical assessments were performed based on values measured preoperatively and every year postoperatively. We performed the Lachman test and anterior translation using a KT-2000 arthrometer (Medmetric, San Diego, CA, USA) at 30° knee flexion with a force of 134 N to evaluate anterior-to-posterior stability. To assess the side-to-side difference for the Lachman test and anterior translation measured using a KT-2000 arthrometer, the values of the affected knee were compared with those of the contralateral non-affected knee. The results of the Lachman test were graded as 0 (<3 mm), 1 (≥3 and <5 mm), 2 (≥5 and <10 mm), or 3 (≥10 mm). Valgus stress radiographs of both knees at 20° knee flexion, obtained using a Telos stress device (Telometer; Daiseung Medics, Seoul, Republic of Korea), were used to evaluate medial instability during the postoperative follow-up examination. With the patient positioned supine and instructed to relax, stress radiography of the knee was performed with a valgus load at the level of the joint line laterally, while two supportive pads were placed on the femur and tibia medially 20 cm from the joint line. On the valgus stress radiograph, we measured the shortest distance between the subchondral bone surface of the most distal medial femoral condyle and the medial tibial plateau [29]. The measured medial joint opening value of the affected knee was compared with that of the contralateral knee, and the side-to-side difference was calculated by subtracting the value of the affected knee from that of the contralateral knee. We did not employ a valgus stress radiograph preoperatively because of the risk of further iatrogenic injury to the MCL. The senior author performed the Lachman test. Anterior translation, measured using a KT-2000 arthrometer, and medial joint opening, measured using valgus stress radiography, were evaluated by two blinded orthopedic fellows, and the mean of the measured values was used.

Functional evaluation was performed for all patients preoperatively and postoperatively using patient-reported outcome measures, including the Lysholm knee scoring scale [30], the International Knee Documentation Committee (IKDC) subjective score [31], and the IKDC examination form [31]. For radiologic assessment of arthritic change, anteroposterior, lateral, and posteroanterior weight-bearing radiographs at 45° knee flexion and Merchant views were taken preoperatively and postoperatively. The IKDC radiographic assessment scale [31] was used for radiological grading.

### 2.3. Statistical Analysis

Statistical analyses were performed using SPSS for Windows (version 26.0; IBM, Armonk, NY, USA). The Shapiro-Wilk test was used to test normality. Depending on the normality, Student’s *t*-test or Mann–Whitney U test was used to compare continuous variables, and the Chi-squared or Fisher exact test was used to compare categorical variables. ANOVA or the Kruskal-Wallis test was used to compare the three groups. Continuous data are reported as the mean ± standard deviation, and categorical data are presented as numbers (percentage). Statistical significance was set at *p* < 0.05. A power analysis was performed using G*Power (version 3.1, Heinrich Heine University Düsseldorf, Düsseldorf, Germany) with a significance level (α) of 5%, based on the mean difference of the side-to-side difference in medial joint opening between the groups [32].

## 3. Results

In Group B, concomitant MCL injuries of grades I, II, and III were noted in 48, 32, and 12 patients, respectively. In Group H, concomitant MCL injuries of grades I, II, and III were observed in 43, 25, and 9 patients, respectively. There were no significant differences between the groups regarding demographic data, including age, sex, affected side, duration from injury to operation, additional diagnosis of meniscal tear treated with partial meniscectomy or meniscal repair, and cartilage lesion grade II or less (Table 1). The proportion of MCL injury grades did not differ between the groups. The preoperative variables between the groups showed no significant differences with regard to the Lachman test, side-to-side difference in anterior translation, Lysholm knee score, IKDC subjective score, IKDC objective grade, and IKDC radiographic grade (Table 2).

**Figure 3:**
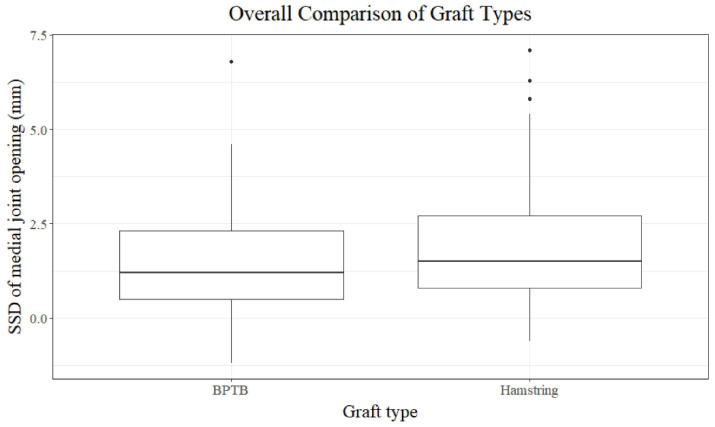
Comparison of the SSD in medial joint opening between the groups. SSD: side-to-side difference; BPTB: bone-patellar tendon–bone.

The postoperative variables, including knee stability, patient-reported outcomes, and radiographic arthritic grade, were not different between the groups after a mean follow-up of 32.1 ± 8.0 months for group B and 33.9 ± 7.9 months for group H (Table 3) (Figure 3). The statistical power calculated based on the side-to-side difference in medial joint opening between the groups was 60.3%.

**Figure 4:**
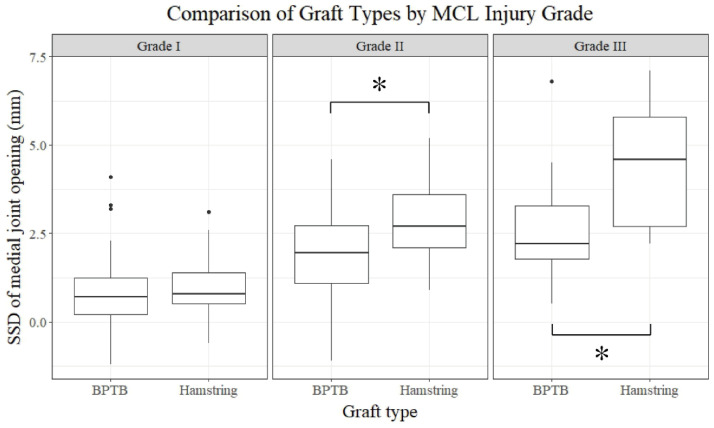
Comparison of the SSD in medial joint opening between the graft types for each MCL injury grade. MCL: medial collateral ligament; SSD: side-to-side difference; BPTB: bone-patellar tendon–bone. An asterisk (*) indicates statistically significant differences (*p* < 0.05).

Comparisons of postoperative values between the types of graft used were performed within each grade of MCL injury. In grade I MCL injuries, there were no significant differences in postoperative values between the groups (*p* > 0.05) (Table 4) (Figure 4). In grade II and III MCL injuries, the side-to-side difference in medial joint opening on the valgus stress radiograph (grade II, *p* = 0.006; grade III, *p* = 0.039) was significantly larger, and the IKDC subjective score (grade II, *p* = 0.045; grade III, *p* = 0.038) was significantly lower for patients using hamstring grafts for ACL reconstruction compared to those using BPTB grafts (Table 5 and Table 6).

**Figure 5:**
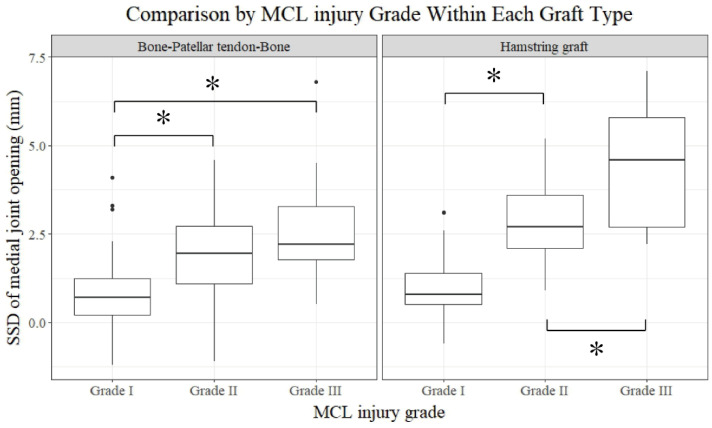
Comparison of SSD in medial joint opening by MCL injury grade within each graft type. MCL: medial collateral ligament; SSD: side-to-side difference. Asterisk (*) indicates statistically significant differences (*p* < 0.05).

Comparisons between the subgroups divided by the grade of MCL injury in groups B and H were conducted. In group B, the side-to-side difference in the medial joint opening gap on the valgus stress radiograph was significantly different between the grades of MCL injury (*p* < 0.001) (Table 7) (Figure 5). However, neither anterior knee instability nor patient-reported outcomes showed significant differences. In group H, patient-reported outcomes, including the Lysholm knee score (*p* = 0.009) and IKDC subjective score (*p* = 0.015), in addition to the side-to-side difference in medial joint opening on valgus stress radiographs (*p* < 0.001), were significantly different between the grades of MCL injuries (Table 8).

## 4. Discussion

The present study primarily aimed to assess the impact of graft choice on the results of ACL reconstruction with non-operatively treated MCL injuries to guide the choice of graft for ACL reconstruction with combined MCL injury. For all combined ACL and MCL injuries without consideration of the MCL injury grade, the choice of the graft did not make a difference in the results of ACL reconstruction regarding knee stability and patient-reported functional outcomes. However, when comparing the graft type according to the MCL injury grade, the use of hamstring grafts for ACL reconstruction in patients conservatively treated for high-grade MCL injuries (grade II or III) resulted in inferior outcomes compared with the use of BPTB grafts in terms of medial knee stability and patient-reported functional outcomes. Furthermore, when comparing postoperative outcomes according to the MCL injury grades among patients who used the same graft type, the Lysholm knee and IKDC subjective scores were inferior for grade III MCL injuries compared to grade I injuries in those who received hamstring grafts, whereas there were no significant differences between the grades of MCL injuries in those who received BPTB grafts. Moreover, medial knee laxity increased as the MCL injury grade increased in the hamstring group.

There is still some controversy regarding the optimal grafts for ACL reconstruction. Since the hamstring and BPTB are the most commonly used grafts for ACL reconstruction, several well-designed studies have compared both grafts [33,34,35,36,37,38,39,40,41]. They showed that both provide similar clinical outcomes regarding knee stability, subjective or objective outcomes, or risk of failure with non-significant or minor differences after ACL reconstruction. However, the optimal graft for ACL reconstruction under the circumstance of non-operatively treated MCL injuries has been rarely investigated, although the favorable results of non-operative management of MCL injuries with ACL reconstruction are well known [8,9,11,12,42]. A recent retrospective registry-based cohort study suggested that the risk of ACL revision and the patient-reported outcome using knee injury and osteoarthritis outcome scores were not different at 2-year follow-up between groups using semitendinosus-gracilis and the patellar tendon as a graft choice in ACL reconstruction with a concomitant non-surgically treated MCL injury, although the grade of MCL injury was not reported [43]. These findings are in agreement with our study, in which the clinical outcomes after ACL reconstruction with non-operatively managed MCL injury regarding knee stability and subjective and objective functional scores were comparable in both the hamstring and BPTB groups.

Regarding comparisons between ACL grafts within the same grade of MCL injury, we found that the use of a hamstring graft negatively influenced medial knee stability and patient-reported functional outcomes, especially in high-grade MCL injuries. In grade II and III MCL injuries, patients who used a BPTB graft had better medial stability measured by the side-to-side difference in medial joint opening on valgus stress radiography and better IKDC subjective scores than those who used a hamstring graft. However, a very cautious approach is required when interpreting these results. The post hoc power analysis for the comparison between the hamstring and BPTB grafts is only 60.3%, which is not high. Therefore, the subgroup analysis could be further underpowered, leading to a risk of increased Type I error, over-interpretation, and inappropriate generalization [44]. To address these issues, additional studies or studies with larger sample sizes are necessary. Nevertheless, we believe that despite these limitations, the data still offer meaningful clinical insights, particularly in identifying trends between the graft types.

Hamstring harvest, especially of the semitendinosus and gracilis muscles, for using ACL grafts could affect the medial knee stability. Several previous biomechanical studies have investigated the effect of hamstring harvest on the clinical result of ACL reconstruction with non-surgically treated MCL injury [24,25,45]. Herbort et al. demonstrated that the semitendinosus and gracilis muscles contributed to the medial stability of the MCL-insufficient knee at a low flexion angle in their human cadaveric study [24]. Another cadaveric investigation simulated ACL reconstruction with concurrent partial MCL injury and concluded that hamstring harvest could lead to increased valgus motion in the setting of ACL reconstruction with partial MCL tear compared to the intact ACL and MCL state [25]. More recently, Vermorel et al., in their cadaveric study, demonstrated that harvesting the medial hamstring tendons not only compromises medial knee stability but also exacerbates medial knee laxity as the severity of MCL injuries increases [45]. In concordance with the previous studies, our study showed that hamstring harvest for the ACL reconstruction graft could negatively affect the medial stability of the knee in contrast to BPTB harvest, particularly in high-grade (II or III) MCL injuries. More pronounced impact in high-grade injuries is likely because grade I injuries, unlike high-grade injuries involving structural disruption, are stretch injuries without any loss of ligamentous integrity, allowing the MCL’s function to mostly recover [46]. Furthermore, we found that hamstring harvest could result in not only worse medial knee stability but also inferior clinical outcomes in high-grade MCL injuries.

Concerning the comparison between the grades of MCL injury within the same type of graft, it seems that a higher grade of MCL injury results in more residual medial knee instability after concurrent ACL reconstruction. The side-to-side difference in medial joint opening on valgus stress radiography was significantly larger in grade III or grade II than in grade I in group B (*p* < 0.05, Table S1). Moreover, medial knee instability measured by the side-to-side difference in medial joint opening on valgus stress radiography increased as the MCL injury grade increased in group H (*p* < 0.001, Table S2). These results suggest that the hamstring harvest aggravated medial instability in addition to the MCL injury itself. Similarly, Zaffagnini et al. compared patients who had undergone ACL reconstruction for isolated ACL injury and combined ACL and grade II MCL injuries. In their prospective evaluation of the 3-year follow-up, valgus laxity was significantly greater in the conservatively treated grade II MCL injured group than in the isolated ACL group [42]. However, the functional outcomes were not different between the groups. Likewise, the functional outcomes in our study were comparable between the grade I and grade II MCL injuries within groups H and B. However, the functional outcomes such as the Lysholm knee score (*p* = 0.010) and IKDC subjective score (*p* = 0.024) were worse in grade III injuries than in grade I injuries within group H, while they were comparable within group B.

Several limitations preclude us from obtaining a definite answer regarding the choice of graft for ACL reconstruction with combined MCL injury. First, the study design was retrospective, and the graft selection for ACL reconstruction was not randomized. As graft selection was based on our own specific criteria rather than randomization, there is a possibility of selection bias that these criteria may have influenced the outcomes. Second, the statistical power was not high (60.3%) based on comparing the mean side-to-side difference in medial joint opening between the groups. Furthermore, the power of the results of the subgroup analyses could have been underpowered owing to the small sample size of some subgroups because the power of the analysis accounted for overall groups instead of subgroups. Future studies with larger sample sizes are necessary to confirm our findings and strengthen the statistical power. Third, the differences in the IKDC subjective scores did not reach a minimum clinically important difference (MCID) of >11.5 [47], although they were statistically significant. Fourth, we excluded the pivot shift test from the evaluation of knee function pre- and postoperatively. Although the pivot shift test is crucial for assessing the rotational stability of an ACL-injured or -reconstructed knee, it could be negative or underestimated in cases of concomitant MCL injury [48].

## 5. Conclusions

The graft choice in ACL reconstruction with concomitant MCL injury may affect clinical outcomes, particularly in high-grade MCL injuries. Although both graft types performed similarly overall, BPTB grafts provided superior medial stability and functional results in higher-grade MCL injuries. However, caution is needed when interpreting these results due to limitations such as the small sample size and the lack of randomization in graft selection.

## Figures and Tables

**Table 1:** Patient demographic data.

Variable	Group B (BPTB Graft) (n = 92)	Group H (Hamstring Graft) (n = 77)	*p*-Value
Age (years) ^a^	33.3 ± 10.3	31.3 ± 8.0	0.148
Sex ^b^			
Male	70 (76.1)	52 (67.5)	0.216
Female	22 (23.9)	25 (32.5)	
Affected side ^b^			
Left	41 (44.6)	34 (44.2)	0.957
Right	51 (55.4)	43 (55.8)	
Duration from injury to operation (weeks) ^a^	8.2 ± 2.5	8.3 ± 2.4	0.596
Additional diagnosis including meniscus and cartilage lesions ^b^			0.891
Yes	54 (58.7)	46 (59.7)	
No	38 (41.3)	31 (40.3)	

^a^ Values are presented as mean ± standard deviation. ^b^ Values are presented as n (%).

**Table 2:** Comparison of preoperative variables between the two groups.

Variable	Group B (BPTB Graft) (n = 92)	Group H (Hamstring Graft) (n = 77)	*p*-Value
Lachman test ^b^			0.702
I	5 (5.4)	5 (6.5)	
II	72 (78.3)	56 (72.7)	
III	15 (16.3)	16 (20.8)	
SSD of Anterior translation ^a,c^	7.8 ± 1.9	7.9 ± 2.0	0.798
MCL injury grade ^b^			0.890
Grade I	48 (52.2)	43 (55.8)	
Grade II	32 (34.8)	25 (32.5)	
Grade III	12 (13.0)	9 (11.7)	
Lysholm knee score ^a^	65.4 ± 17.3	67.8 ± 10.8	0.914
IKDC subjective score ^a^	51.7 ± 14.1	52.7 ± 8.7	0.577
IKDC objective grade ^b^			0.745
A	0	0	
B	4 (4.3)	4 (5.2)	
C	69 (75.0)	54 (70.1)	
D	19 (20.7)	19 (24.7)	
IKDC radiographic grade ^b^			>0.999
A	87 (94.6)	73 (94.8)	
B	5 (5.4)	4 (5.2)	
C	0	0	
D	0	0	

SSD: side-to-side difference; IKDC: International Knee Documentation Committee. ^a^ Values are presented as mean ± standard deviation. ^b^ Values are presented as n (%). ^c^ Measured with a KT-2000 arthrometer at 30° knee flexion with a force of 134 N.

**Table 3:** Comparison of postoperative variables between the two groups.

Variable	Group B (BPTB Graft) (n = 92)	Group H (Hamstring Graft) (n = 77)	*p*-Value
SSD of medial joint opening ^a,c^	1.4 ± 1.4	2.0 ± 1.7	0.060
Lachman test ^b^			0.949
0	71 (77.2)	58 (75.3)	
I	15 (16.3)	14 (18.2)	
II	6 (6.5)	5 (6.5)	
SSD of anterior translation ^a,d^	2.3 ± 1.3	2.6 ± 1.5	0.317
Lysholm knee score ^b^	85.2 ± 8.9	85.0 ± 7.5	0.576
IKDC subjective score ^b^	83.5 ± 6.6	81.6 ± 8.2	0.110
IKDC objective grade ^b^			0.419
A	68 (73.9)	51 (66.2)	
B	18 (19.6)	17 (22.1)	
C	6 (6.5)	9 (11.7)	
D	0	0	
IKDC radiographic grade ^b^			0.722
A	81 (88.0)	66 (85.7)	
B	11 (12.0)	10 (13.0)	
C	0	1 (1.3)	
D	0	0	

SSD: side-to-side difference; IKDC: International Knee Documentation Committee. ^a^ Values are presented as mean ± standard deviation. ^b^ Values are presented as n (%). ^c^ Measured from a valgus stress radiograph using a Telos stress device at 20° knee flexion. ^d^ Measured using a KT-2000 arthrometer at 30° knee flexion with a force of 134 N.

**Table 4:** Comparison of postoperative values between two subgroups divided according to the selected graft material in grade I MCL injuries.

Variable	Grade I MCL Injury (n = 91)		*p*-Value
	BPTB (n = 48)	Hamstring (n = 43)	
SSD of medial joint opening ^a,c^	0.9 ± 1.0	0.9 ± 0.8	0.479
Lachman test ^b^			0.787
0	39 (81.3)	33 (76.7)	
I	7 (14.6)	7 (16.3)	
II	2 (4.2)	3 (7.0)	
SSD of anterior translation ^a,d^	2.1 ± 1.2	2.4 ± 1.4	0.511
Lysholm knee score ^a^	85.8 ± 9.8	87.2 ± 7.2	0.690
IKDC subjective score ^a^	83.0 ± 6.6	83.7 ± 7.0	0.640
IKDC objective grade ^b^			0.591
A	36 (75.0)	29 (67.4)	
B	10 (20.8)	10 (23.3)	
C	2 (4.2)	4 (9.3)	
D	0	0	
IKDC radiographic grade ^b^			>0.999
A	43 (89.6)	39 (90.7)	
B	5 (10.4)	4 (9.3)	
C	0	0	
D	0	0	

SSD: side-to-side difference; IKDC: International Knee Documentation Committee. ^a^ Values are presented as mean ± standard deviation. ^b^ Values are presented as n (%). ^c^ Measured from a valgus stress radiograph using a Telos stress device at 20° knee flexion. ^d^ Measured using a KT-2000 arthrometer at 30° knee flexion with a force of 134 N.

**Table 5:** Comparison of postoperative values between two subgroups divided according to the selected graft material in grade II MCL injuries.

Variable	Grade II MCL Injury (n = 57)		*p*-Value
	BPTB (n = 32)	Hamstring (n = 25)	
SSD of medial joint opening ^a,c^	1.9 ± 1.4	2.9 ± 1.3	**0.006**
Lachman test ^b^			>0.999
0	24 (75.0)	19 (76.0)	
I	6 (18.8)	5 (20.0)	
II	2 (6.3)	1 (4.0)	
SSD of anterior translation ^a,d^	2.2 ± 1.3	2.4 ± 1.4	0.658
Lysholm knee score ^a^	85.1 ± 7.5	83.6 ± 5.9	0.430
IKDC subjective score ^a^	84.4 ± 6.4	80.1 ± 9.3	**0.045**
IKDC objective grade ^b^			>0.999
A	24 (75.0)	19 (72.0)	
B	6 (18.8)	5 (20.0)	
C	2 (6.3)	2 (8.0)	
D	0	0	
IKDC radiographic grade ^b^			0.720
A	28 (87.5)	21 (84.0)	
B	4 (12.5)	4 (16.0)	
C	0	0	
D	0	0	

SSD: side-to-side difference; IKDC: International Knee Documentation Committee. ^a^ Values are presented as mean ± standard deviation. ^b^ Values are presented as n (%). ^c^ Measured from a valgus stress radiograph using a Telos stress device at 20° knee flexion. ^d^ Measured using a KT-2000 arthrometer at 30° knee flexion with a force of 134 N.

**Table 6:** Comparison of postoperative values between two subgroups divided according to the selected graft material in grade III MCL injuries.

Variable	Grade III MCL Injury (n = 21)		*p*-Value
	BPTB (n = 12)	Hamstring (n = 9)	
SSD of medial joint opening ^a,c^	2.7 ± 1.7	4.4 ± 1.9	**0.039**
Lachman test ^b^			>0.999
0	8 (66.7)	6 (66.7)	
I	2 (16.7)	2 (22.2)	
II	2 (16.7)	1 (11.1)	
SSD of anterior translation ^a,d^	3.0 ± 1.5	3.8 ± 1.9	0.286
Lysholm knee score ^a^	83.2 ± 8.6	78.1 ± 8.1	0.187
IKDC subjective score ^a^	82.9 ± 7.3	75.9 ± 6.8	**0.038**
IKDC objective grade ^b^			0.605
A	8 (66.7)	4 (44.4)	
B	2 (16.7)	2 (22.2)	
C	2 (16.7)	3 (33.1)	
D	0	0	
IKDC radiographic grade ^b^			0.591
A	10 (83.3)	6 (66.6)	
B	2 (16.7)	2 (22.2)	
C	0	1 (11.1)	
D	0	0	

SSD: side-to-side difference; IKDC: International Knee Documentation Committee. ^a^ Values are presented as mean ± standard deviation. ^b^ Values are presented as n (%). ^c^ Measured from a valgus stress radiograph using a Telos stress device at 20° knee flexion. ^d^ Measured using a KT-2000 arthrometer at 30° knee flexion with a force of 134 N.

**Table 7:** Comparison of postoperative values between three subgroups divided according to the MCL injury grades in Group B (BPTB graft).

Variable	BPTB Graft (n = 92)			*p*-Value
	Grade I Injury (n = 48)	Grade II Injury (n = 32)	Grade III Injury (n = 12)	
SSD of medial joint opening ^a,c^	0.9 ± 1.0	1.9 ± 1.4	2.7 ± 1.7	**<0.001**
Lachman test ^b^				0.532
0	39 (81.3)	24 (75.0)	8 (66.7)	
I	7 (14.6)	6 (18.8)	2 (16.7)	
II	2 (4.2)	2 (6.3)	2 (16.7)	
SSD of anterior translation ^a,d^	2.1 ± 1.2	2.2 ± 1.3	3.0 ± 1.5	0.112
Lysholm knee score ^a^	85.8 ± 9.8	85.1 ± 7.5	83.2 ± 8.6	0.506
IKDC subjective score ^a^	83.0 ± 6.7	84.4 ± 6.4	82.9 ± 7.3	0.634
IKDC objective grade ^b^				0.626
A	36 (75.0)	24 (75.0)	8 (66.7)	
B	10 (20.8)	6 (18.8)	2 (16.7)	
C	2 (4.2)	2 (6.3)	2 (16.7)	
D	0	0	0	
IKDC radiographic grade ^b^				0.743
A	43 (89.6)	28 (87.5)	10 (83.3)	
B	5 (10.4)	4 (12.5)	2 (16.7)	
C	0	0	0	
D	0	0	0	

SSD: side-to-side difference; IKDC: International Knee Documentation Committee. ^a^ Values are presented as mean ± standard deviation. ^b^ Values are presented as n (%). ^c^ Measured from a valgus stress radiograph using a Telos stress device at 20° knee flexion. ^d^ Measured using a KT-2000 arthrometer at 30° knee flexion with a force of 134 N.

**Table 8:** Comparison of postoperative values between three subgroups divided according to the MCL injury grade in Group H (hamstring graft).

Variable	Hamstring Graft (n = 77)			*p*-Value
	Grade I Injury (n = 43)	Grade II Injury (n = 25)	Grade III Injury (n = 9)	
SSD of medial joint opening ^a,c^	0.9 ± 0.8	2.9 ± 1.3	4.4 ± 1.9	**<0.001**
Lachman test ^b^				0.829
0	33 (76.7)	19 (76.0)	6 (66.7)	
I	7 (16.3)	5 (20.0)	2 (22.2)	
II	3 (7.0)	1 (4.0)	1 (11.1)	
SSD of anterior translation ^a,d^	2.4 ± 1.4	2.4 ± 1.4	3.8 ± 1.9	0.094
Lysholm knee score ^a^	87.2 ± 7.2	83.6 ± 5.9	78.1 ± 8.1	**0.009**
IKDC subjective score ^a^	83.7 ± 7.0	80.1 ± 9.3	75.9 ± 6.8	**0.015**
IKDC objective grade ^b^				
A	29 (67.4)	18 (72.0)	4 (44.4)	
B	10 (23.3)	5 (20.0)	2 (22.2)	
C	4 (9.3)	2 (8.0)	3 (33.3)	
D	0	0	0 (0)	
IKDC radiographic grade ^b^				0.097
A	39 (90.7)	21 (84.0)	6 (66.7)	
B	4 (9.3)	4 (16.0)	2 (22.2)	
C	0	0	1 (11.1)	
D	0	0	0	

SSD: side-to-side difference; IKDC: International Knee Documentation Committee. ^a^ Values are presented as mean ± standard deviation. ^b^ Values are presented as n (%). ^c^ Values are presented as n (%). ^d^ Measured using a KT-2000 arthrometer at 30° knee flexion with a force of 134 N.

## Data Availability

Data are available upon request to the corresponding author.

## Supplementary Materials

The following supporting information can be downloaded at: https://www.mdpi.com/article/10.3390/jcm13216316/s1, Table S1: Pairwise comparison of postoperative values between the three subgroups divided according to MCL injury grade in Group B (bone-patellar tendon-bone graft); Table S2: Pairwise comparison of postoperative values among the three subgroups divided according to MCL injury grade in Group H (hamstring graft).
