# Supplementary material for: Effect of Graft Choice for ACL Reconstruction on Clinical Outcomes in Combined ACL and MCL Injuries: Comparison Between Bone-Patellar Tendon-Bone and Hamstring Autografts
Source: J Clin Med. 2024 Oct 22;13(21):6316. doi: 10.3390/jcm13216316 (PMC11546250; doi:10.3390/jcm13216316)
Supplement: Supplementary file 1 [file jcm-13-06316-s001.zip › jcm-3240927-supplementary.pdf]

Table S1

Pairwise comparison of postoperative values between three subgroups divided according to MCL injury grade in Group B (bone-patellar tendon-bone graft)

		SSD of medial joint opening[a]
MCL injury grade	Grade I vs. Grade II	**0.001**
	Grade II vs. Grade III	0.687
	Grade I vs. Grade III	**0.001**

SSD: Side-to-side difference

[a]The p-values according to the pairwise comparisons are presented.

Table S2

Pairwise comparison of postoperative values between three subgroups divided according to MCL injury grade in Group H (hamstring graft)

		SSD of medial joint opening[a]	Lysholm knee score[a]	IKDC subjective score[a]
MCL injury grade	Grade I vs. Grade II	**< 0.001**	0.310	0.214
	Grade II vs. Grade III	**0.002**	0.254	0.506
	Grade I vs. Grade III	**< 0.001**	**0.010**	**0.024**

SSD: Side-to-side difference; IKDC: International Knee Documentation Committee

[a]The p-values according to the pairwise comparisons are presented.
